# Antibiotics treatment promotes vasculogenesis in the brain of glioma-bearing mice

**DOI:** 10.1038/s41419-024-06578-w

**Published:** 2024-03-13

**Authors:** Maria Rosito, Javeria Maqbool, Alice Reccagni, Ottavia Giampaoli, Fabio Sciubba, Fabrizio Antonangeli, Ferdinando Scavizzi, Marcello Raspa, Federica Cordella, Lucrezia Tondo, Silvia Di Angelantonio, Flavia Trettel, Alfredo Miccheli, Giuseppina D’Alessandro, Cristina Limatola

**Affiliations:** 1 Department of Physiology and Pharmacology, Sapienza University, Rome, Italy; 2 Center for Life Nanoscience & Neuroscience Istituto Italiano di Tecnologia@Sapienza, Rome, Italy; 3 Department of Environmental Biology, Sapienza University, Rome, Italy; 4 NMR-Based Metabolomics Laboratory (NMLab), Sapienza University, Rome, Italy; 5 Institute of Molecular Biology and Pathology, National Research Council (CNR), Rome, Italy; 6 EMMA CNR, Monterotondo, Italy; 7 IRCCS Neuromed, Pozzilli, IS Italy; 8 Department of Physiology and Pharmacology, Sapienza University, Laboratory Affiliated to Institute Pasteur Italia, Rome, Italy

**Keywords:** CNS cancer, Microglia, Tumour angiogenesis, Metabolomics

## Abstract

In recent years, several studies described the close relationship between the composition of gut microbiota and brain functions, highlighting the importance of gut-derived metabolites in mediating neuronal and glial cells cross-talk in physiological and pathological condition. Gut dysbiosis may affects cerebral tumors growth and progression, but the specific metabolites involved in this modulation have not been identified yet. Using a syngeneic mouse model of glioma, we have investigated the role of dysbiosis induced by the administration of non-absorbable antibiotics on mouse metabolome and on tumor microenvironment. We report that antibiotics treatment induced: (1) alteration of the gut and brain metabolome profiles; (2) modeling of tumor microenvironment toward a pro-angiogenic phenotype in which microglia and glioma cells are actively involved; (3) increased glioma stemness; (4) trans-differentiation of glioma cells into endothelial precursor cells, thus increasing vasculogenesis. We propose glycine as a metabolite that, in ABX-induced dysbiosis, shapes brain microenvironment and contributes to glioma growth and progression.

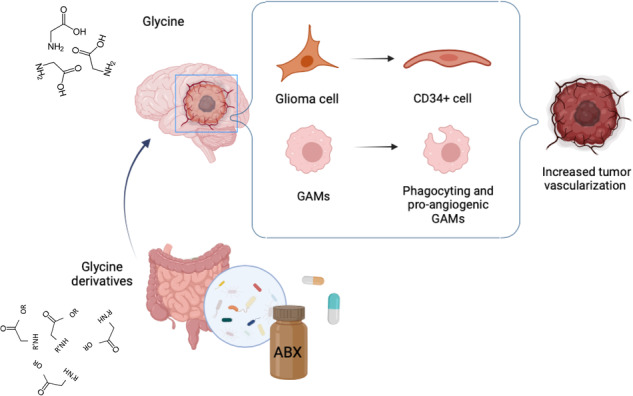

## Introduction

Recently, several studies demonstrated that the gut microbiota and microbiota-derived metabolites impact on brain function both in physiological and pathological conditions [1, 2]. Microglia cells sense the brain microenvironment and modify their functions regulating brain homeostasis or contributing to brain disfunction, also in the context of brain tumor [3]. It has been demonstrated that gut dysbiosis also interferes with microglial functions, affecting the interaction with synapses in healthy mice [4–7], and altering the cross talk with astrocytes in pathological conditions [8, 9]. In the context of brain tumor, we have previously shown that the gut dysbiosis induced by antibiotics (ABX) treatments, in glioma-bearing mice, promotes tumor growth and impairs the natural killer (NK) cells effector functions [10]. The effects of ABX treatment are mediated by a massive selection of the microbiota that colonizes the intestinal tract and the consequent alteration of the metabolites produced. Thus, it is relevant to understand the signature molecules that orchestrate the crosstalk among the enteric system, the brain and the tumor under normal conditions and upon ABX administration.

Recently, mass spectrometry-based metabolomics has provided significant support in the identification and detection of the diverse range of molecules produced by the human microbiota [11–14]. Microbe-derived factors such as bacterial structural proteins, molecules associated with microbes, short-chain fatty acids and neurotransmitters play both local and systemic functions in various processes. These include the maintenance of the blood-brain barrier [15], of the gut epithelial integrity [16], the activation and the maturation of microglia [4, 7], the regulation of the immune response and inflammation in different neurodegenerative diseases [5, 17], the modulation of brain plasticity [18] and the growth of brain tumors [18, 19].

Hallmarks of glioblastoma biology include high invasiveness of tumor cells and the presence of abundant and aberrant vasculature [20]. In the context of brain tumors, glioma-associated microglia/macrophages (GAMs) establish close interactions with tumor cells, playing a pivotal role in tumor growth and progression [3, 21]. In addition to exerting an immunosuppressive role [22–24], it has been reported that GAMs are actively involved in the processes of tumor angiogenesis [25–27].

The formation of new vessels in glioma relies on different sophisticated biological strategies involving the proliferation and migration of endothelial cells from the host endothelium [28] or supported by the mobilization and integration of endothelial progenitor cells [29, 30], whose circulating levels are elevated in glioblastoma patients [31, 32].

In addition to these strategies, glioma stem cells (GSC) can differentiate toward mesenchymal lineage cell types [33]. Similar to neural stem cells, GSCs have the ability to self-renewal, to differentiate into multiple cell lineage, and to participate in vessel formation. Consequently, through interactions with the vascular niche, GSCs play a crucial role in shaping the tumor microenvironment (TME) and can differentiate into endothelial-like tumor cells, contributing to the development of a vascular network structure.

In this study we found that chronic treatment with non-absorbable ABX, namely gentamicin and vancomycin, impacts on GAMs behaviors in a syngenic GL261 mouse model. Peritumoral microglial cells demonstrated an impaired ability to rearrange their processes in response to ATP stimulation and GAMs (identified as CD11b^+^ cells) acquired a pro-angiogenic phenotype.

Our findings revealed that ABX treatment modulated the mice metabolomic profile in the gut as well as in the brain, enhanced tumor vascularization and increased the frequency of the CD133^+^CD34^+^ cells population within the tumoral core. Among the modulated metabolites, we identified glycine as one putative mediator of the ABX-induced pro-phagocytic and angiogenic microglia phenotype, and of the trans-differentiation of tumor cells toward a CD133^+^CD34^+^ phenotype.

## Results

### ABX treatment affects GAMs phenotype and impairs ATP-induced response

**Fig. 1:**
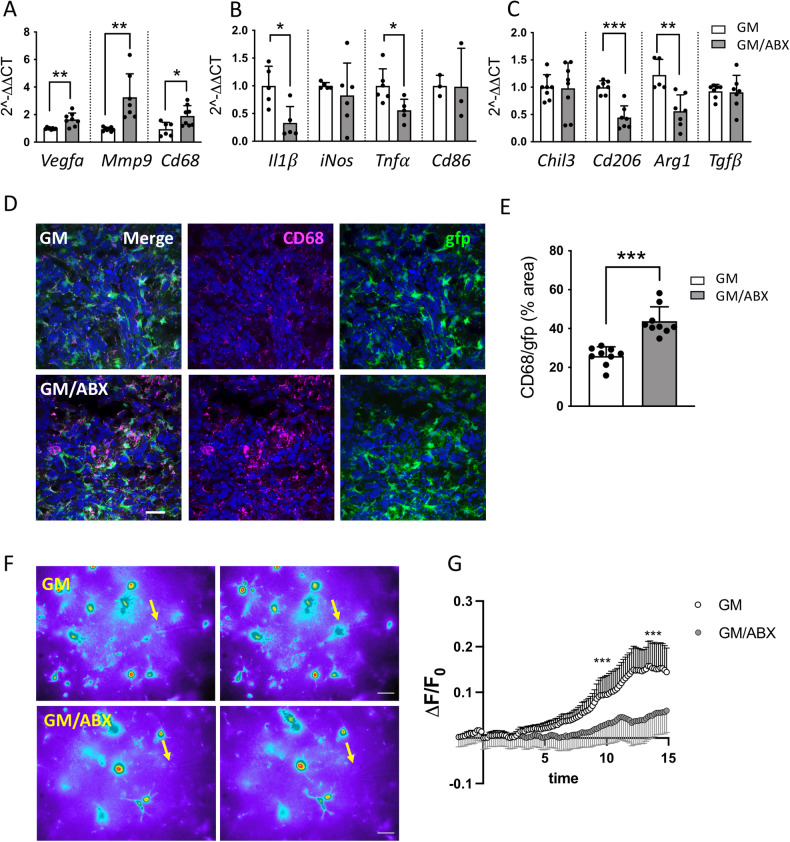
Tumor-associated microglia/macrophages phenotyping and functional properties. **A** RT-qPCR from CD11b^+^ cells isolated from GM and GM/ABX tumoral hemisphere reveals expression of pro-angiogenic genes; **B** pro-inflammatory genes and (**C**) anti-inflammatory genes. Gene expression is normalized to the housekeeping gene Gapdh. Data are presented as the mean ± SD *n* = 3–8 mice pulled from 3 independent experiments. ***p < 0.001; **p < 0.01; *p < 0.05, Student’s *t* test. **D** Representative z-projection confocal images of microglia/macrophages cells in the tumoral core area from GM and GM/ABX Cx3cr1^+/gfp^ mice (green) and CD68 (magenta). Hoechst staining (blu) for nuclei visualization. 60x objective (Scale bar: 25 μm). **E** Scatter dot plots showing quantification of CD68/gfp signals (from Cx3cr1^+/gfp^ mice) expressed as the percentual area occupied in GM (*n* = 33/9/3 FOV/slices/mice) and GM/ABX (*n* = 38/9/3 FOV/slices/mice) mice versus total field of view (FOV). Data are presented as the mean ± SD ***p < 0.001, Student’s *t* test. **F** Representative fields of GFP fluorescence measurements in slices from GM and GM/ABX Cx3cr1^+/gfp^ mice at minute 0 (left) and after 15 min (right) of ATP. The arrows refer to the tip of the puff pipette. After 5 min of basal motility recordings, ATP is applied for 15 min (Mg-ATP 2 mM, 8 psi, 100 ms). Note the fluorescence increase in the area around the pipette tip only in control slices. Scale bar: 10 µm. **G** Time course of fluorescence ratio signal (∆*F*/*F*0) measured in a ROI of 10 µm radius, centered on the tip of the ATP-containing pipette, from slices of GM and GM/ABX-treated Cx3cr1^+/gfp^ mice. (GM *n* = 8/4 fields/mice GM/ABX *n* = 11/4 fields/mice; Data are presented as the mean ± SD ***p < 0.001, Mann–Whitney test at minutes 10 and 15).

Gut dysbiosis may impact microglia function [4, 6] and tumor growth [10]. To investigate how gut dysbiosis affects microglial cells in the context of glioma, mice were treated with two not orally absorbable ABX (vancomycin and gentamicin) for 2 weeks and then brain transplanted with GL261 glioma cells (GM) (Fig. S1A). Three weeks later, upon continuous ABX administration, CD11b^+^ cells were isolated from the ipsilateral tumoral hemisphere and analyzed for gene expression by RT-PCR. ABX treatment increases the expression of the pro-angiogenic genes VEGFα and MMP9 and of the phagocytic marker CD68 (Fig. 1A), while reduces the expression of genes involved in inflammation (IL1β, TNFα, Arg1 and CD206) (Fig. 1B, C). The increased expression of CD68 was also confirmed by immunofluorescence staining in ABX-treated GL261 bearing Cx3cr1^+/gfp^ mice (Fig. 1D, E).

To further investigate the effect of ABX treatment on microglia function, we analyzed microglia patrolling activity in the peritumoral area in response to a focal ATP source [34] (Fig. 1F). Microglial ability to extend processes toward ATP was assessed by time-lapse acquisition in GM and GM/ABX brain slices from Cx3cr1^+/gfp^ mice. With this procedure, we visualized the displacement of microglia processes toward the ATP source through increased fluorescence around the tip of ATP-releasing pipet. Data shown in Fig.1G demonstrate that ABX treatment reduces the speed of process movement toward ATP, indicative of an ABX-dependent impairment of microglial function as previously observed in mice treated with ABX [6]. According to our previous results [10], we validated the impact on glioma growth, thus confirming a significant increase of tumor volume in GM/ABX-treated mice in comparison with GM mice (GM 9.57 ± 6.7, GM/ABX 16.74 ± 9.4 mm^3^ *p = 0.031) (Fig. S1B) and confirmed an increased tumor volume upon ABX treatment also in Cx3cr1^+/gfp^ mice (GM 5.48 ± 3.0, GM/ABX 8.79 ± 3.7 mm^3^ *p = 0.025) (Fig. S1C). We also confirmed that there are no differences in the frequency of microglia (CD45^+^CD11b^+^TMEM119^+^) between the tumor-bearing hemisphere of control and ABX-treated mice (Fig. S1D).

### ABX-induced dysbiosis enhances glioma vasculogenesis

Since in our experimental condition ABX treatment induced a pro-angiogenic and tissue remodeling phenotype in CD11b^+^ cells, we analyzed the effect of ABX on glioma vascularization.

**Fig. 2:**
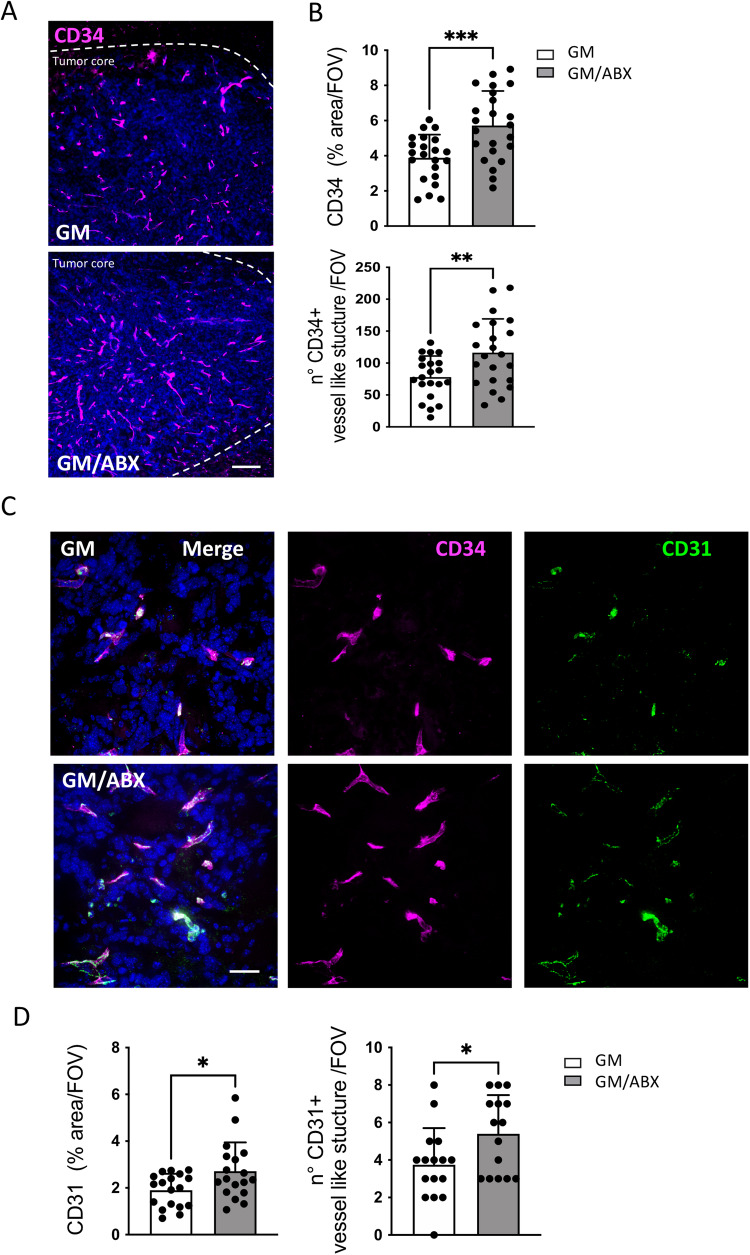
Vasculature characterization in GM and GM/ABX mice. **A** Representative z-projection confocal images of CD34 (magenta) in GM and GM/ABX tumor core area. Hoechst staining (blu) for nuclei visualization Scale bar: 50 μm. **B** Scatter dot plots showing quantification of CD34 signal expressed as the percentual area occupied by fluorescent CD34 staining in GM (*n* = 22/5 slices/mice) and GM/ABX (*n* = 23/5 slices/mice) mice versus total FOV (top) and number of CD34^+^ vessel-like structure versus total field of view (bottom) GM (*n* = 22/5 slices/mice) and GM/ABX (*n* = 23/5 slices/mice). Data are presented as the mean ± SD ***p < 0.001; **p < 0.01, Student’s *t* test. **C** Representative z-projection confocal images of CD34 (magenta) and CD31 (green) colocalization in GM and GM/ABX tumor core area. Hoechst staining (blu) for nuclei visualization Scale bar: 20 μm. **D** Scatter dot plots showing quantification of CD31 signal expressed as the percentual area occupied by fluorescent CD34 staining in GM (*n* = 18/3 slices/mice) and GM/ABX (*n* = 18/3 slices/mice) mice versus total field of view (left) and number of CD31^+^ vessel-like structure versus total field of view (GM *n* = 18/3 slices/mice; GM/ABX *n* = 15/3 slices/mice) (right). Data are presented as the mean ± SD *p < 0.05, Student’s *t* test.

At this aim we characterized the expression of CD34^+^ in GL261 bearing mice; this endothelial marker plays pivotal roles in the development of glioma and influences tumor progression [35, 36]. Immunofluorescence staining revealed that ABX-treated mice exhibit higher reactivity for CD34 in the tumoral hemisphere (Fig. 2A, B top) and increased expression of CD34^+^ vessel-like structures compared to control mice (GM 78.05 ± 33; GM/ABX 116.4 ± 53, number of structure/Field of view (FOV); **p = 0.0071) (Fig. 2B, bottom), thus suggesting an increased vasculogenesis.

Double immunofluorescence staining revealed that the CD34^+^ vessel-like structures are also positive for the endothelial marker CD31 (Fig. 2C). ABX-treated mice exhibit an increased signal density for CD31 (Fig. 2D, left), and an increased number of CD31^+^ vascular structures (GM/ABX 5.4 ± 2.0, GM 3.7 ± 1.9 number of structure/FOV; *p = 0.05) (Fig. 2D, right).

Similar results were obtained with mice transplanted with a different glioma cell line, CT-2A; also in this syngeneic murine glioma model, ABX treatment induced an increase in tumor volume (GM 49.6 ± 11.7, GM/ABX 62.5 ± 8.8 mm^3^; *p = 0.033) (Fig. S2A) and increased tumor vasculogenesis in comparison to control mice (GM 6.88 ± 1.6, GM/ABX 8.125 ± 1.4% of area/FOV; *p = 0.03) (Fig. S2B, C).

### ABX treatment increases the expression of the CD34 endothelial marker on glioma cells

**Fig. 3:**
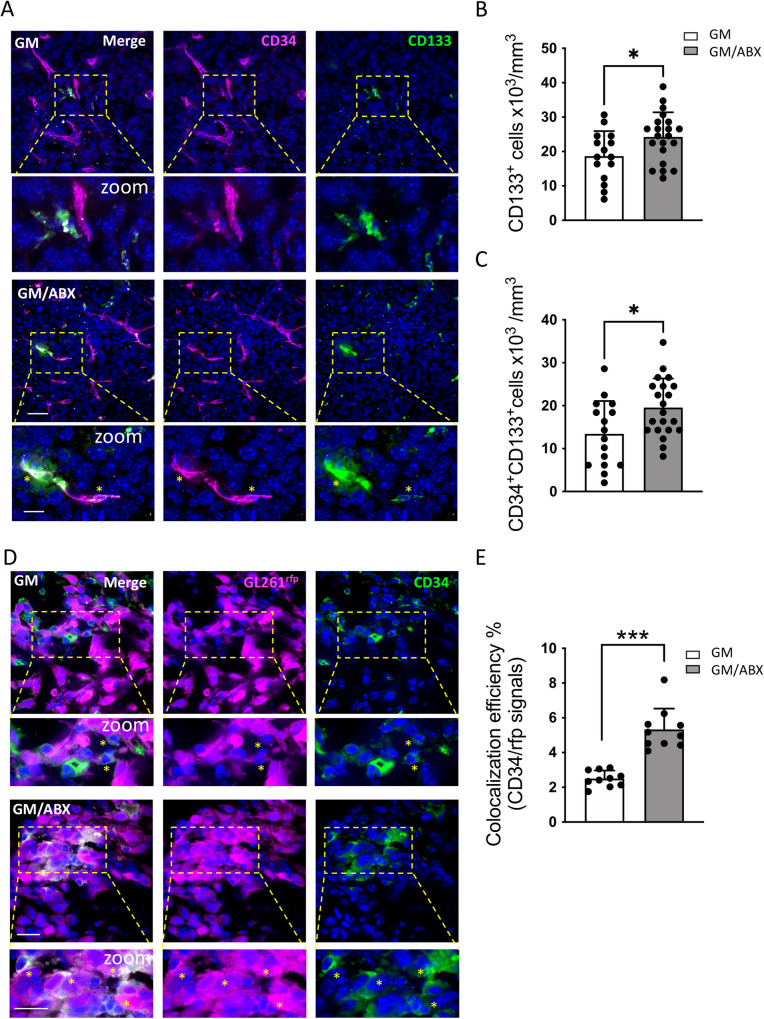
Characterization of CD34^+^ cells in glioma tissue. **A** Representative z-projection confocal images of CD34 (magenta) and CD133 (green) in GM and GM/ABX tumor core area. Hoechst staining (blu) for nuclei visualization Scale bar: 25 μm; zoom 10 μm. **B** Scatter dot plots showing quantification of CD133^+^ positive cells (GM: *n* = 16/7/3 FOV/slices/mice; GM/ABX: *n* = 21/8/3 FOV/slices/mice. Data are presented as the mean ± SD *p < 0.05; Student’s *t* test). **C** Scatter dot plots showing quantification of CD133^+^ CD34^+^ double-positive cells (GM: *n* = 16/7/3 FOV/slices/mice; GM/ABX: *n* = 21/8/3 FOV/slices/mice. Data are presented as the mean ± SD *p < 0.05; Student’s *t* test). **D** Representative single-plane confocal images of GL261^rfp^ (magenta) and CD34 (green) in GM and GM/ABX tumor core area. Hoechst staining (blu) for nuclei visualization. 60x objective (Scale bar: 20 μm; zoom 20 μm). Yellow stars indicate the colocalizing signals. **E** Scatter dot plots showing the percentage of rfp-CD34 colocalizing signals (GM *n* = 40/10/3 FOV/slices/mice; GM/ABX *n* = 40/10/3 FOV/slices/mice). Data are presented as the mean ± SD ***p < 0.001 Student’s *t* test.

Several mechanisms have been described to drive the process of vasculogenesis in glioma [37, 38]: both GSCs and cells of hematopoietic origin display the ability to undergo endothelial cell differentiation [39–41]. We therefore investigated the effect of ABX administration on the expression of CD133 marker in the mice tumor-bearing hemisphere. The quantification of CD133^+^ cells revealed that GM/ABX mice exhibit a higher number of GSCs in the tumoral core (Fig. 3A, B) (GM 18.67 ± 7.2, GM/ABX 24.24 ± 7.1 cells × 10^3^/mm^3^; *p = 0.028). Double immunofluorescence staining shows that the CD133^+^ cells exhibit an endothelial differentiation profile as revealed by the co-expression of the CD34 endothelial marker (Fig. 3A); and that GM/ABX mice display in the tumor core a higher number of CD133^+^CD34^+^ cells compared to GM mice (Fig. 3C) (GM 13.41 ± 7.7, GM/ABX 19.57 ± 6.8 cells × 10^3^/mm^3^; *p = 0.014). These results suggest that ABX treatment increases the abundance of the CD133^+^CD34^+^ cells population in the tumoral area.

In order to investigate the origin of these vessel-like structures, mice were inoculated with GL261^rfp^ cells, and tissue sections from GM-transplanted mice were stained for CD34 to identify GL261 cells expressing the endothelial marker. Confocal immunofluorescence analysis describes that GM/ABX mice exhibit higher GL261^rfp+^ CD34^+^ colocalization signals with respect to GM mice in the tumor core area (Fig. 3D, E) (GM 2.5 ± 0.5, GM/ABX 5.33 ± 1.2 colocalization efficiency %; ***p < 0.001). These data demonstrate that ABX treatment promotes the GL261 trans-differentiation toward the endothelial phenotype.

### Endothelial progenitor cells infiltration is not modulated by ABX treatment

**Fig. 4:**
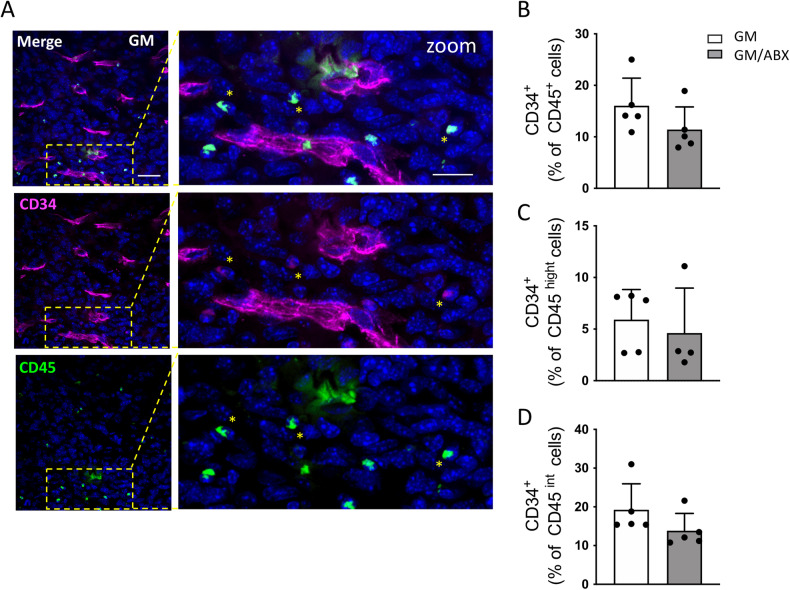
Characterization of CD45^+^CD34^+^ cells in glioma tissue. **A** Representative z-projection confocal images of CD34 (magenta) and CD45 (green) in the GM tumor core area. Hoechst staining (blu) for nuclei visualization. 60x objective (Scale bar: 25 μm; zoom 20 μm). Yellow stars indicate the CD45^+^CD34^+^ cells. **B** Flow cytometry quantification of CD45^+^CD34^+^ cells in GM and GM/ABX tumoral hemisphere. **C** CD45^high^CD34^+^ population identifying the peripheral infiltrating hematopoietic endothelial progenitor cells and (**D**) CD45^int^CD34^+^ identifying the resident brain population expressing the CD34 marker (mostly likely microglia/macrophages). *n* = 5 mice per group. Data are presented as the mean ± SD.

Since the formation of new vessels in tumors can be supported by the mobilization and the integration of circulating endothelial progenitor cells that are present in glioma [29, 30], we investigated the presence of CD45^+^CD34^+^ hematopoietic progenitors in tumoral brain hemisphere. By immunofluorescence, we detected the presence of CD45^+^CD34^+^ double-positive cells (Fig. 4A) that can be identified in the tumoral core as a cell population distinct from the CD45^−^CD34^+^ vessel-like structure. We then investigated, by flow cytometry analysis, the frequency of CD34^+^ cells among the total CD45^+^ cells (GM 16.04 ± 5.3, GM/ABX 11.41 ± 4.4% of cells; *p* = 0.17; *n* = 5 animals per group (Fig. 4B), discriminating the peripheral infiltrating (CD45^high^) (GM: 5.91 ± 2.9; GM/ABX: 4.67 ± 4.3% of cells; *p* = 0.38; *n* = 5 animals per group; Fig. 4C) from the resident (CD45^int^) cells (GM: 19.24 ± 6.7; GM/ABX: 13.84 ± 4.5% of cells; *p* = 0.17; *n* = 5 animals per group; Fig. 4D). Our data demonstrated that ABX treatment does not affect the abundance of CD45^+^CD34^+^ cells.

### Glycine induces TME and glioma cells to adopt a pro-angiogenic phenotype

It has been demonstrated that long-term ABX treatment compromises the composition and functions of the gut microbiota inducing long-lasting detrimental effects on the host. In order to evaluate the perturbations induced by 2 weeks ABX treatment, ^1^H NMR metabolomic analysis has been carried out on fecal waters and brain extracts of these mice. In Fig. S4A, spectrum of fecal water is reported and total of 33 metabolites have been quantified by NMR spectroscopy (Supplementary Table 1). From the comparison of CTRL (untreated mice) and ABX spectra (Fig. S4B), we observed that the ABX treatment has determined the almost total disappearance of the shorth chain fatty acids (butyrate, propionate, acetate) signals, while signals of choline and molecule attributed to a glycine derivative (Gly-derivative) based on ^1^H-^13^C Heteronuclear Single Quantum Correlation (HSQC) and Heteronuclear Multiple Bond Correlation (HMBC) experiments, were much more intense in ABX, with a 32-fold and 83-fold average increase respectively. Furthermore, the fecal water spectra of ABX-treated mice exhibited a series of broad, unresolved bands between 3 ppm and 5.5 ppm, signals attributed to gentamicin and vancomycin metabolites and therefore not included in the quantitative analysis and statistical discussion.

**Fig. 5:**
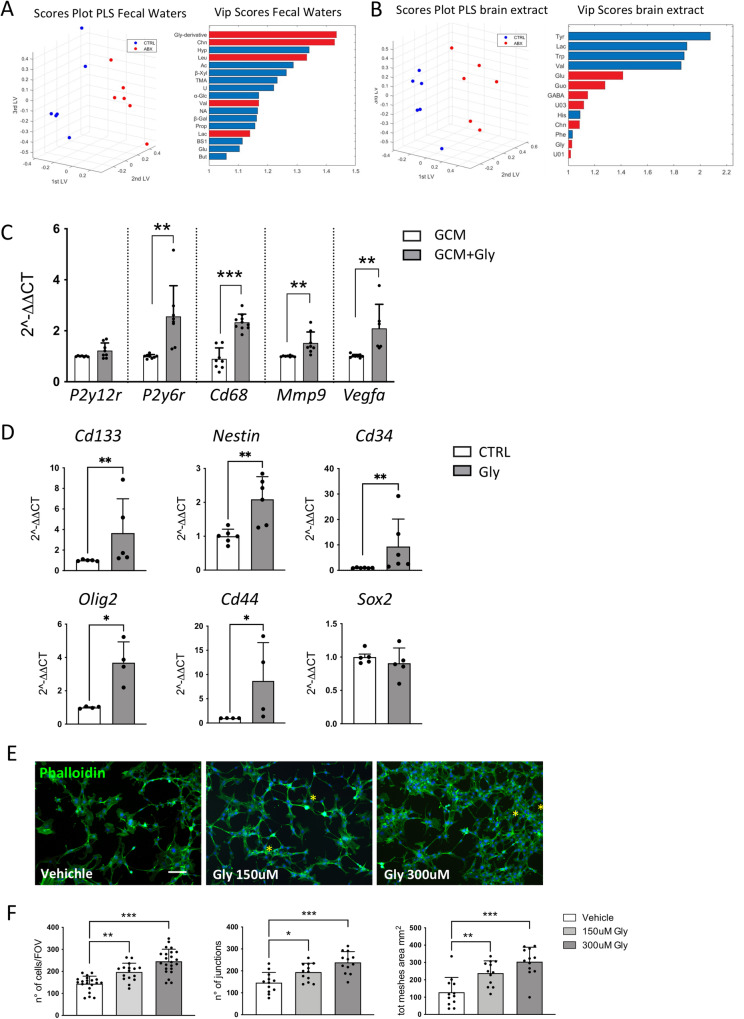
Glycine effect on microglia, GL261 and murine endothelial cells. **A** PLS scores plot (left) and VIP scores (right) of fecal waters from CTRL (blue) and ABX-treated mice (red). **B** PLS scores plot (left) and VIP scores (right) of brain extracts from CTRL (blue) and ABX-treated mice (red). VIP values are reported in horizontal histograms and values of VIP higher than 1 are statistically significant. Metabolite that is higher in ABX is reported in red, while the metabolite that is lower in ABX is reported in blue. **C** RT-qPCR from microglia mRNA. Gene expression is normalized to the housekeeping gene Gapdh, *n* = 4–6 independent experiments. Data are presented as the mean ± SD ***p < 0.001; **p < 0.01; Student’s *t* test. **D** RT-qPCR from GL261 mRNA. Gene expression is normalized to the housekeeping gene Gapdh, *n* = 4–6 independent experiments. Data are presented as the mean ± SD **p < 0.01; *p < 0.05, Student’s *t* test. **E** Representative images of bEnd.3 cells labeled with Alexa Fluor 488-conjugated phalloidin (green) and Hoechst for nuclei visualization (blue). Yellow asterisk indicates junctions and meshes wall generated in cell culture upon glycine treatment. Scale bar: 25 μm. **F** Plot showing the number of cells counted per field of view (FOV) (Left), the number of junctions (middle), and total meshes area (right) in the in vitro cell angiogenesis assay. (Vehicle *n* = 20, 150 μM glycine *n* = 16, and 300 μM *n* = 23 FOV from 3 independent experiments). Data are presented as the mean ± SD ***p < 0.001; **p < 0.01; *p < 0.05. One-way ANOVA—Dunnett’s multiple comparison test.

We then applied the partial least squares (PLS) model to the data matrix. The optimal complexity of the PLS model, as assessed by full cross-validation, was found to be three latent variables (LVs), which explained the 74.47% and 99.39% of the total variance on x and y, respectively. The validation results were *R*^2^ = 0.99, *Q*^2^ = 0.97 and ROC = 0.99 (Fig. 5A, left) for correctly classifying ABX from CTRL. Seventeen metabolites were mostly responsible for the discrimination, in particular Gly-derivative, Choline (Chn), Leucine (Leu), Valine (Val), Lactate (Lac) were higher and Hypoxanthine (Hyp), Acetate (Ac), beta-Xylose (beta-Xyl), Trimethylamine (TMA), Uracil (Ura), alpha-Glucose (alpha-Glc), Nicotinate (NA), beta-Galactose (beta-Gal), Propionate (Prop), Bile salts 1, Glutamate (Glu) and Butyrate (But) were significantly lower after ABX treatment (Fig. 5A, right).

In brain extracts, thirty-nine molecules have been identified and quantified by ^1^H-NMR and were also included for six CTRL and six ABX-treated mice (Supplementary Table 2). A representative spectrum of brain is also reported in Fig. S4C.

NMR-based metabolomics of murine cerebral tissue showed a different profile after ABX treatment compared to the CTRL group. As depicted by the PLS score plot in Fig. 5B (left), the first three LVs explained the 24.5% on x and 66.7% on y of the total variance, showing a *R*^2^ = 0.67, *Q*^2^ = 0.42 and ROC = 0.68. Thirteen variables were significantly involved in the separation of the groups as indicated by the variable importance of projection (VIP) scores >1 (Fig. 5B right). In particular, Glutamate (Glu), Guanosine (Guo), γ-aminobutyric acid (GABA), (Chn), Glycine (Gly), Unknown 01 (U01) and Unknown 03 (U03) were higher in ABX, while the levels of Valine (Val), Tyrosine (Tyr), Lac, histidine (His), tryptophan (Trp), phenylalanine (Phe) were lower in ABX compared to CTRL.

We focused our attention on glycine since previous studies demonstrated that this aminoacid has pro-angiogenic activities [42, 43] and that GL261 cells express the intracellular glycine receptor GlyRa1 [44]. We first investigated whether glycine could affect microglial cell phenotype in the context of glioma. To this aim, RT-PCR analysis was performed on primary microglial cells cultured in GL261 conditioned medium (GCM) in the presence or in the absence of glycine (300 mM), for 72 h. Data reported in Fig. 5C show that glycine-treated microglia have an increased expression of P2y6r, Cd68, Mmp9, and Vegfα, all genes related to phagocytosis and angiogenesis (**p < 0.01; ***p < 0.001).

Moreover, we analyzed the effect of glycine on cultured GL261 cells and observed an increased expression of stemness-related genes such as Cd133, Nestin, Cd34, Olig2, and Cd44 (Fig. 5D) (*p < 0.05; **p < 0.01). No variation of Sox2 transcripts was observed. Glycine administration did not affect GL261 cell viability (MTT assay) and proliferation (BrdU assay) as reported in Fig. S5A, B.

Co-option of the pre-existing vessels in the neighboring tissue is one of the most critical steps utilized by the tumor to support the metabolic and nutrient needs [45]. We thus investigated whether glycine treatment could establish an angiogenic-like model in endothelial cells and reported that glycine-treated bEND3 cells show increased cell proliferation, cell sprouting, and connections, as shown by the formation of a complex mesh of cells forming polygonal structures [46, 47] (Fig. 5E, F). All these data show that ABX treatment results in the increased level of several factors in the brain including glycine. The latter, acting on different cellular targets, promotes stemness and angiogenesis.

### Glycine promotes glioma growth and vasculogenesis in ABX-treated mice

In order to validate the in vivo effect of glycine in promoting tumor growth and vasculogenesis, we tested the effect of a selective and orally active glycine transporter 1 (GlyT1) inhibitor [48–50]. It has been demonstrated that GlyT1 inhibitor is able to increase the plasma and cerebrospinal fluid glycine concentration in rats and humans [50, 51].

**Fig. 6:**
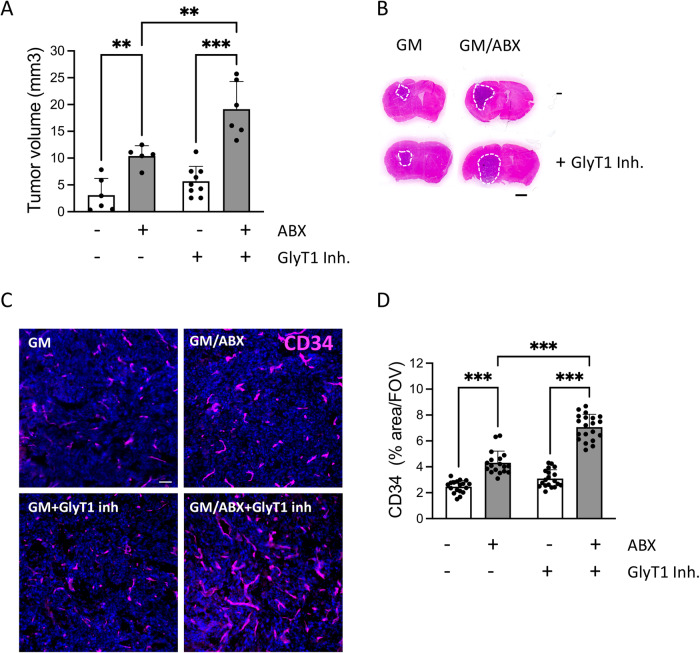
In vivo effect of GlyT1 inhibitor on tumor volume and vasculogenesis. **A** Tumor size in GM and GM/ABX mice with or without GlyT1 inhibitor, *n* = 5–9, pooled from two experiments. Data are presented as the mean ± SD **p < 0.01, ***p < 0.001, one-way ANOVA followed by Tukey’s multiple comparison test. **B** Representative images of brain coronal slices, scale bar = 1 mm. **C** Representative confocal images of CD34 (magenta) in GM and GM/ABX tumor core area. Hoechst staining (blu) for nuclei visualization Scale bar: 50 μm. **D** Scatter dot plots showing quantification of CD34 signal expressed as the percentual area occupied by fluorescent CD34 staining in GM (*n* = 18/3 slices/mice), GM/ABX (*n* = 18/3 slices/mice), GM + GlyT1 inh (*n* = 19/3 slices/mice), GM/ABX + GlyT1 inh (*n* = 20/3 slices/mice) mice versus total field of view (FOV) Data are presented as the mean ± SD ***p < 0.001; one-way ANOVA followed by Tukey’s multiple comparison test.

We analyzed the tumor volume in GM and GM/ABX mice treated or not with GlyT1 inhibitor: the treatment is able to significantly increase the tumor volume in GM/ABX mice versus GM mice (GM + GlyT1 inh. 5.68 ± 2.8, GM/ABX + GlyT1 inh. 19.14 ± 5.14 mm^3^ ***p < 0.001) and further increases the ABX effect on tumor growth (GM/ABX 10.39 ± 1.9, GM/ABX + GlyT1 inh. 19.14 ± 5.14 mm^3^ **p < 0.01) (Fig. 6A, B). Moreover the immunofluorescence staining for vessel-like structure characterization revealed that GM/ABX mice treated with the GlyT1 inhibitor exhibit a higher reactivity for CD34 in the tumoral core versus GM mice (GM + GlyT1 inh. 3.1 ± 0.1, GM/ABX + GlyT1 inh. 7.06 ± 0.2% of area/FOV; ***p < 0.001) and that GlyT1 inhibitor treatment boost the vasculogenic effect mediated by ABX administration (GM/ABX 4.3 ± 0.2, GM/ABX + GlyT1 inh 7.06 ± 0.2% of area/FOV; ***p < 0.001) (Fig. 6C, D). All together these results show that glycine is a mediator of glioma growth and vasculogenesis in ABX-treated mice.

## Discussion

The gut microbiota and metabolome have been reported to play key roles in the regulation of several processes related to brain functions [18, 52, 53] and host disease pathogenesis [54, 55]. Dysbiosis in the gut can be caused by several factors such as imbalanced diets, infections, environmental stressors and use of drugs, all affecting gut microbial diversity and establishing the conditions for host susceptibility to diseases [56]. In recent years a close relationship between the gut microbiome and microglial functions has been reported [4–7], and we had previously demonstrated that long-term ABX administration alters the gut microbiota composition and increases glioma growth in mice [10]. We here investigated the metabolic alterations induced by a prolonged treatment with the non-absorbable ABX gentamicin and vancomycin, and described the effects on the TME. Further, we identified glycine as a possible mediator of the pro-angiogenic TME phenotype induced by ABX treatment on glioma bearing mice.

It has been reported that GAMs are involved in the process of tumor angiogenesis, with a preferential role for resident microglia [25]. Here we did not discriminate among the different cell populations, but described that CD11b^+^ cells, isolated from the tumoral hemisphere of ABX-treated mice, exhibit a pro-angiogenic and phagocytic phenotype as revealed by the increased transcriptional level of Vegfα, Mmp9 and Cd68, markers associated with vasculogenesis, tissue remodeling and tumor progression [57–59].

We also described that in the context of glioma, ABX treatment induced an impairment of microglial phenotype, as demonstrated by a reduced transcription of some pro-inflammatory and anti-inflammatory genes, and by an impaired ability to respond to local ATP variation, a common homeostatic response against brain perturbations [60–63]. All these findings are in line with the altered microglia phenotype observed in germ-free and antibiotics or probiotic treated mice [4, 64].

Glioblastoma, like other solid tumors, relies on blood supply by specialized vascular structures to meet the increased metabolic demands [39, 41, 65, 66] and vasculature, in brain tumors, correlates with their aggressive phenotype [65, 66]. In the complex glioblastoma landscape, several mechanisms drive vasculogenesis: (1) the recruitment of circulating endothelial progenitor cells into the vascular architecture of brain tumor [67, 68]; (2) the lining of tumor vessels and tumor cells [68]; (3) a process of transdifferentiation where cancer stem-like cells differentiate into endothelial cells [69]. Here we reported that GM/ABX-treated mice exhibit increased CD34^+^CD31^+^ vascular-like structure in the tumor core. Moreover, the increased abundance of CD133^+^CD34^+^ cell population suggests that the process of vasculogenesis may rely on one or more stem cell populations inside the tumor area. In addition, we found that ABX treatment increases the expression of CD34 marker on GL261^rfp^ cells, suggesting glioma cells transdifferentiation into an endothelial progenitor phenotype.

The increased vasculogenesis could also depend on infiltrating hematopoietic CD34^+^ stem cells [68]; upon ABX treatment, however no evidence of alteration in the abundance of CD45^+^CD34^+^ cells was observed thus suggesting that in our experimental conditions, there is no contribution of blood-derived endothelial progenitor cells to vasculogenesis.

In order to identify putative mediators that can contribute to ABX-induced glioma growth and tumor vasculogenesis, we took advantage of the metabolomic analysis of fecal and brain extracts by ^1^H NMR, and identified glycine and its derivatives as metabolites associated with ABX treatment in glioma bearing mice.

Glycine is an aminoacid which plays essential roles in many biological processes, including immunomodulation [70], neurotransmission [71] and angiogenesis [42], both in physiological and pathological conditions. Glycine affects microglial phenotype in an in vivo model of brain ischemia [72] and modulates phagocytosis in the BV2 microglial cell line [73, 74]. We described that primary cultures of microglial cells respond to glycine treatment increasing the expression of P2y6 receptor and Cd68, both associated to phagocytosis, and increasing the expression of Mmm9 and Vegfα, involved in tissue remodeling and angiogenesis.

The role of glycine has been investigated on different tumors [75, 76]. In glioma, glycine facilitates tumor progression acting on its intracellular receptor GlyRa1, and GlyRa1 silencing reduces glioma volume in mice [44]. More recently, glycine has been proposed as a biomarker of glioma subtypes and grades, being abundant in IDH mutant and in glioblastoma [77, 78].

We demonstrate that glycine administration to GL261 cells increases the expression of the stemness-related gene CD133, without affecting cell proliferation. Cancer stem cells have been characterized in glioma and play important roles in driving tumor growth [79, 80]. It has been shown that CD133^+^ GL261 cells generate gliomaspheres with high proliferative index [81, 82].

We also observed that glycine increased the frequency of CD34^+^ CD133^+^ GL261 cells, indicating an effect on glioma cell transdifferentiation toward an endothelial phenotype. Moreover, the increased transcriptional level of oligodendrocyte transcription factor 2 (OLIG2) suggests that GL261 trans-differentiation is linked to multiple strategies of tumor vasculature production and remodeling. It has been shown that Olig2^+^ oligodendrocyte precursor-like glioma cells are involved in the process of tumor vasculogenesis [83]. Additional GSCs markers increased upon glycine treatment are CD44, a large cell adhesion molecule that interacts with the extracellular matrix components [84] and positively correlated with tumor invasion and progression [85, 86], and nestin. The high expression of nestin has been correlated to increased glioma proliferation and is a bona fide marker of angiogenesis [87, 88]. Glycine function in the angiogenesis process has been also described by Guo et al. [42], in a mouse model of hindlimb ischemia, where glycine administration promoted neovascularization and recovery of vascular flow. In accordance, here we also found that glycine improves the endothelial cell network in the bEnd3 cell line.

We have studied the role of glycine in ABX-treated mice, taking advantage of a GlyT1 selective inhibitor. Our results show that increased level of glycine in the brain further boost the ABX effect on glioma growth and vascularization. The effect on glioma growth is in line with what reported by Förstera et al. [44], that showed how the knock down of the intracellular glycine receptor (GlyR1) in GL261 impairs tumor progression. In ABX-induced dysbiosis, the brain metabolic profile is altered and, in this specific brain microenvironment, glycine is fueling glioma growth.

In conclusion, here we report that ABX-induced gut dysbiosis affects the glioma TME. Among the putative metabolites potentially involved in driving such effect, we highlight the role of glycine that, acting on microglial, glioma and endothelial cells, establishes a pro-angiogenic environment in the brain tumor area thus leading tumor progression. These data support the determinant role of the gut-brain cross-talk in glioma and provide evidence that modulation of gut microbiota and metabolome may impact on glioma vasculogenesis.

## Materials and methods

### Animal husbandry

C57BL/6N mice were housed (two to four animals per cage) under a 12-h light cycle in standard cages, in autoclaved bedding and drinking water, and with sterilized standard chow ad libitum, and were randomly assigned to the different experimental groups. For CD68 immunofluorescence analysis and time-lapse imaging, Cx3cr1^+/gfp^ mice were used (crossing B6.129P2(Cg)-Cx3cr1tm1Litt/J from Jackson Laboratory with C57BL6J). All the experiments in this study were conducted in accordance with the ARRIVE guidelines [89], and were approved by the local animal welfare body and by the Italian Ministry of Health (authorization No. 231/2015PR) in accordance with the EC Council Directive 2010/63/EU and the Italian d.lgs.26/2014. All the efforts were done to minimize animal suffering, and to reduce the number of animals, calculating the necessary sample size before starting the experiments.

### Tumor cells transplantation and ABX treatment

Glioma syngeneic mouse model (GM) are obtained injecting glioma cells (GL261; CT2-a and GL261-rfp) as previously described [10]. Mice were treated with not-absorbable ABX (0.5 g/l vancomycin, AppliChem; [0.5 g/l], gentamicin, Aurogene) and sucralose (0.5%) to improve palatability or with sucralose alone (control solution) in autoclaved water. ABX and control solutions were changed every 2 days, and continuously administrated for 2 weeks before and 3 weeks after tumor injection.

### Drug administration

GlyT1 inhibitor (BI-425809, Iclepertin) is administered by oral gavage to the mice (2.5 mg/Kg, 0,3% DMSO), drug is sonicated for 2 min soon before the oral gavage. Mice are treated every 4 days starting from the beginning of ABX administration and throughout the experimental setup. Mice that didn’t receive the pharmacological treatment were subjected to oral gavage with the same volume of vehicle solution.

### Tumor volume evaluation

Serial 20 μm coronal brain slices (one every 100 μm of the entire tumor length) were collected and stained by standard H&E protocol. Tumor volume was calculated according to the formula (volume = *t* ×$\sum$ *A*), where *A* is the tumor area/slice and *t* is the thickness by ImageTool3.0 software.

### Isolation of CD11b^+^ cells and extraction of total RNA

Glioma bearing mice were anesthetized and perfused with PBS. Brains were removed, the ipsilateral brain hemisphere was cut into small pieces and single-cell suspension was achieved in Hank’s balanced salt solution (HBSS). The tissue was further mechanically dissociated using a glass wide-tipped pipette and the suspension was applied to a 30 μm cell strainer (Miltenyi Biotec). Cells were processed immediately for MACS MicroBead separation. CD11b^+^ cells were magnetically labeled with CD11b MicroBeads. The cell suspension was loaded onto a MACS Column placed in the magnetic field of a MACS Separator and the negative fraction was collected. After removing the magnetic field, CD11b^+^ cells (which contain resident microglial cells but also infiltrating monocytes/macrophages, granulocytes and natural killer cells) were eluted as a positive fraction. Total RNA was isolated with Tri-Reagent (Merck), and processed for real-time PCR. The quality and yield of RNAs were verified using the NANODROP One system (Thermo Scientific).

### Slice preparation

Acute coronal brain slices were prepared from CTRL and ABX-treated Cx3cr1^+/gfp^ mice [90] injected with GL261 cells, in chilled artificial cerebrospinal fluid (ACSF). The ACSF composition included the following concentrations in millimolar (mM): NaCl 125, KCl 2.3, CaCl_2_ 2, MgCl_2_ 1, NaHPO_4_ 1, NaHCO_3_ 26 and glucose 10. To maintain physiological pH, the ACSF was continuously oxygenated with a mixture of 95% O_2_ and 5% CO_2_. Using a slicer (7000 smz-2 Vibratome, Campden Instruments, UK), coronal slices with a thickness of 250 μm were cut at 4 °C. Slices were then placed in a chamber filled with oxygenated ACSF and allowed to recover at room temperature (24–25 °C) for at least 2 h. All time lapse recordings were conducted at room temperature on the submerged slices in ACSF. The recording chamber was perfused with the same ACSF solution at a rate of 1 ml/min while being observed under a microscope.

### Time-lapse imaging in acute brain slices

Microglia process rearrangement in acute striatal slices was assessed using time-lapse fluorescence [34, 91]. Throughout the experiment, the slices were continuously maintained in oxygenated ACSF at room temperature. Images were captured every 10 s for a duration of 50 min, with an exposure time of 200 ms, using an Olympus BX51WI microscope equipped with a LUMPlanF N 40×/0.80 (water immersion) objective (Olympus Corporation, Tokyo, JP). To excite the GFP signal at 488 nm, an Optoscan monochromator (Cairn Research, Faversham, UK) was utilized. The light source was a xenon lamp Optosource (Cairn Research). A borosilicate glass micropipette filled with ACSF supplemented with 2 mM Mg-ATP (Merck) was positioned at the core of the recording field, within the peritumoral area, using a Sutter Instruments micromanipulator MP-225 (Novato, CA, USA). The pipette was placed ~50 µm beneath the surface of the slice to avoid reactive microglia.

The basal fluorescence was assessed for 5 min, then a small volume of Mg-ATP solution was applied to the recording field core using a pneumatic pico-pump (PV820; World Precision Instruments, Inc., Sarasota, FL, USA) with a brief pressure (8 psi; 100 ms) [92]. Images were acquired using a Photometrics CCD CoolSnap MYO camera (Tucson, AZ, USA) and analyzed using MetaFluor software. Fluorescence variations were measured in five concentric circular regions of interest (ROIs) positioned from the tip of the ATP pipette, with diameters of 10, 20, 40, 80, and 120 µm. The fluorescence signal was determined using the formula (*F* − *F*_0_)/*F*_0_, where *F*_0_ represents the average fluorescence before ATP application.

### Immunofluorescence staining on brain tissue and image acquisition

Mice were anesthetized with isoflurane 3%, and transcardially perfused with cold PBS and 4% PFA in 0.1 M PB. Brains were rapidly removed, postfixed overnight in PFA 4%, washed with PB, and cryoprotected in PB 30% sucrose solution. We then collected 20 μm-thick coronal sections with a cryostat microtome (Leica Microsystems) at 20 °C. Briefly, slices were immersed for 30 min in a boiling citrate buffer solution for antigen retrieval, then incubated with blocking solution (0.5% Triton X-100, 5% BSA) for 1 h at RT. Sections were incubated with primary antibodies (CD34, RAM-34 Thermo Fisher, 1:100; CD31, 28364 Abcam, 1:100; CD133, PA538014 Thermo Fisher, 1:100; CD45 PA585429 Thermo Fisher 1:100; GFAP MAB30060 Millipore 1:300; CD68 FA-11 eBioscience 1:100) in diluted blocking solution overnight at 4 °C and 1 h at RT with fluorophore-conjugated secondary antibodies (Alexa Fluor 488 goat anti-rabbit, 594 goat anti-mouse; Alexa Fluor 488 goat anti-rat; Alexa Fluor 594 goat anti-rat) and Hoechst for nuclei visualization. The sections were mounted with a fluorescence mounting medium (DAKO) or with ProLong Glass Antifade Mountant (Thermo Fisher).

Images were collected with spinning disk confocal microscopy on an Olympus IX73 microscope equipped with X-Light V3 spinning disk (CrestOptics), LDI laser source and a Prime BSI Scientific CMOS (sCMOS), 6.5 µm pixels (Photometrics) with a 60x/1.4 PlanApo l oil objective and 10x objectives. The used Z step size was 1 µm and 0.5 µm respectively for 10X and 60X objectives. All the images were acquired by using Metamorph software version 7.10.2. (Molecular Devices, Wokingham, UK) and then analyzed with ImageJ or Metamorph software (see Image preparation and analysis).

### Image preparation and analysis

For image preparation, we used the open-source software ImageJ for adjustments of levels and contrast, maximum intensity projections, and thresholding signals for fluorescence intensity analysis. For vessel-like structure analysis, stack images were flattened in a maximum intensity 10 um z-projection, and after removing the background noise, images were binarized allowing the count of the vessel-like structure identified considering a threshold of 100 um^2^. For angiogenesis analysis, we used the angiogenesis analyzer plugin from ImageJ.

For GL261^rfp^–CD34^+^ colocalization study, after image thresholding, the single-plane confocal acquisition was analyzed by quantifying the percentage of colocalizing regions using Metamorph software.

### Cell isolation and flow cytometric analysis

The study was conducted in accordance with the reported flow cytometry guidelines [93]. Single-cell suspensions were obtained from GM and GM/ABX tumoral hemispheres. Mice were intracardially perfused with PBS and brains were rapidly removed, hemispheres separated, and placed into ice-cold HBSS. The hemispheres were disrupted in a glass-Teflon homogenizer and passed through a 100 μm nylon cell strainer (Becton-Dickinson). The suspension was centrifuged (800 × *g*, 10 min, RT), and the pellet was resuspended in 8 ml of 30% Percoll (Sigma) and overlaid on the top of HBSS. The suspension was centrifuged (14,000 × *g*, 15 min, RT), the pellet was washed with 10% FBS in HBSS, and cells used for flow cytometry or labeled with CD11b^+^ microbeads and passed through MACS Columns (Miltenyi Biotec). For flow cytometry, cells were washed and suspended in a staining buffer (PBS with 0.5% BSA, 2 mM EDTA, 0.025% NaN3). Zombie Violet™ Fixable Viability Dye from BioLegend was used to exclude dead cells and anti-CD16/32 (clone 2.4G2) was used to prevent nonspecific and Fc-mediated binding. Cells were stained with the following fluorochrome-conjugated mAbs (clone name indicated in parentheses) for 20 min at 4 °C: CD45.2-APC-eFluor 780 (104), CD11b-APC (M1/70), Tmem119-Alexa Fluor™ 488 (V3RT1GOsz) from eBioscience TM-Invitrogen (Thermo Fisher), CD34-PE (RAM-34) from eBioscience^TM^. Samples were analyzed by a FACS-CantoII (BD Biosciences), and data were elaborated using FlowJo software v.10.7.1 (TreeStar, Ashland, OR, USA). Gating strategies are shown in Supplementary Figs. 2 and 3A.

### Sample preparation for NMR analysis

Brain samples were rapidly frozen in liquid nitrogen, weighted and immediately extracted with a modified Bligh-Dyer protocol, using 2:2:1 ratio of chloroform, methanol and water, respectively. After 24 h at 4 °C, the samples were centrifuged at 11,000 × *g* for 25 min. The top hydrophilic fraction (methanol-water) was then collected to a scintillation vial and then dried under nitrogen flux. Each sample have been subsequently resuspended in 0.7 ml of D_2_O solution containing the internal standard 3-(trimethylsilyl)-propionic-2,2,3,3-d_4_ acid sodium salt (TSP, 2 mM).

Frozen stools were combined with 1.2 ml of phosphate buffer (PBS)-D_2_O-NaN_3_ (0.5% v/v). Samples were thawed for 30 min at 25 °C and then vortexed to achieve a homogenous solution. The supernatant was separated from the solid phase through a first centrifugation at 10,000 × *g* for 25 min at 4 °C, filtered on a 40 μm pores filter. After adding 200 μl of PBS-D_2_O with 0.3% of NaN_3_, the samples were centrifuged again at 10,000 × *g* for 25 min at 4 °C. In total, 600 µl of supernatants were added to 60 µl of PBS-D_2_O-TSP (2 mM, final concentration).

### NMR acquirement and processing

#### ^1^H experiments

The NMR ^1^H monodimensional spectra were recorded at 25 °C on a JEOL ECZR JNM spectrometer operating at the proton frequency of 600.13 MHz. Spectra were acquired collecting 128 scansions for each sample using a calibrated 90° detection pulse length of 8.3 µs, 64k data points and a spectral width of 15 ppm. Presaturation has been employed for water signal suppression and the relaxation delay have been set to 7.723 s, in order to achieve a 15 s of total acquisition time, guaranteeing complete resonance relaxation between following scansions.

Spectra have been processed by applying an exponential window function with a line broadening factor LB = 0.3 Hz. After applying the Fourier transformation, spectra have been manually phased and base corrected by applying the BCFR protocol. Metabolites quantitation has been carried out by comparing the integrals of specific resonances with the one of the internal standards and normalized by the number of protons. Data from brain and liver have been expressed as µmol/g, for fecal water metabolites concentration as nmol/g.

#### Bidimensional experiments

To univocally identify the metabolites in the biological samples, bidimensional experiments ^1^H-^1^H Total Correlation Spectroscopy (TOCSY) and ^1^H-^13^C Heteronuclear Single Quantum Correlation (HSQC) and Heteronuclear Multiple Bond Correlation (HMBC) were performed on selected samples. TOCSY experiments were conducted with a spectral width of 9025 Hz in both dimensions, a data matrix of 8192 × 256 points, a mixing time of 80 ms, and a relaxation delay of 2 s. HSQC experiments were performed with spectral widths of 9025 KHz and 37,764 KHz for the proton and carbon, respectively, a data matrix of 8192 × 256 points and a recycle delay of 2 s. HMBC experiments have been acquired with a spectral width of 9025 KHz and 37,764 KHz for the proton and carbon, respectively, with a data matrix of 8 K × 256 points, long-range constants nJC–H of 4, 8, and 12 Hz, and a recycle delay of 3 s.

### Microglia primary cultures and cell treatment

Microglia cells are obtained as previously described [94]. In detail, cortical glial cells were prepared from 0- to 2-day-old mice: cerebral cortices were chopped and digested in 30 U/ml papain for 40 min at 37 °C followed by gentle trituration. The dissociated cells were washed, suspended in DMEM with 10% FBS (Invitrogen) and 2 mM L-glutamine, and plated at a density of 9–10 × 10^5^ in 175 cm^2^ cell culture flasks. At confluence (10–14 DIV), glial cells were shaken for 2 h at 37 °C to detach and collect microglial cells. After seeding, cells were treated for 48 h with Glycine (300 μM) in the presence of glioma conditioned medium (GCM). GCM is collected from GL261 (1 × 10^6^ cells in 1 ml of DMEM -FBS).

### Cell lines and treatments

The GL261 murine glioma cells (provided by Dr. Michela Matteoli Humanitas Milan) were cultured in DMEM supplemented with 20% FBS. Glycine was purchased from MERCK, and treatment (300 μM) was provided in Minimum Essential Medium (w/o FBS) for 72 h. After stimulation cells were harvested and total RNA was isolated with Tri-Reagent (Merck) and processed for real-time PCR. The quality and yield of RNAs were verified as above. bEnd.3 cells were seeded in DMEM 10% FBS at the density of 10^4^ cells/cm^2^, stimulated with glycine (150–300 μM) and after 8 h treatment cells were fixed and stained with phalloidin for vessel-like structure analysis (see “Image analysis” section).

### MTT assay

After glycine treatments, MTT (dissolved in PBS with a final density of 0.5 mg/ml) was added to the medium culture. After 2 h incubation, the MTT solution was extracted, mixed with DMSO, and shaken for 20 min. Finally, the absorption of the samples was read by regulating the 570-nanometer filter as the main wavelength and the 630-nanometer filter as the referenced wavelength. Blank was subtracted from all samples to obtain pure cellular absorption.

### BrdU cell immunostaining

GL261 cells were grown on glass coverslips at a density of 5 × 10^4^ cells/cm^2^ and treated for 6 days with Glycine 300 µm or vehicle. Cells were then incubated with 10 μg/ml BrdU for 30 min, washed with PBS, and fixed in 4% paraformaldehyde for 15 min. Fixed cells underwent immunostaining protocols as described for brain sections. Hoechst was used for nuclear staining. BrdU-positive cells were counted out of 800 cells for condition.

### Real-time PCR

Samples were lysed in TRYzol reagent for isolation of total RNA. The quality and yield of RNAs were verified using NANODROP One (Thermo Scientific). For RT-PCR one microgram of total RNA was reverse transcribed using ThermoScript RT-PCR System. RT-PCR was carried out using Sybr Green (Bio-Rad) according to the manufacturer’s instructions. The PCR protocol consisted of 40 cycles of denaturation at 95 °C for 30 s and annealing/extension at 60 °C for 30 s. For quantification, the comparative Threshold Cycle (Ct) method was used. The Ct values from each gene were normalized to the Ct value of GAPDH in the same RNA samples. Relative quantification was performed using the 2^−∆∆Ct^ method and expressed as fold change in arbitrary values. Oligos used for gene expression are listed in Supplementary Table 3.

### Statistical analysis

The repeat (*n*) for each experiment and details of statistical analyses are described in the figure legends or main text. Data are reported as mean ± SD. Statistical analysis, normality tests and non-parametric tests were performed, when appropriate, with GraphPad Prism 9 software. The exact *p* values are indicated in the text where available and the multiplicity-adjusted *p* values are indicated in the corresponding figures (*p < 0.05, **p < 0.01, ***p < 0.001). Paired *T*-test is used to compare tumor volume and FACS analysis; an unpaired *T*-test is used for immunofluorescence analysis. For real-time PCR a Mann–Whitney *U* test was run to determine significant differences for the considered genes.

The in vitro angiogenesis assay (number of cells, junction, and total meshes area) was analyzed by one-way ANOVA followed by Dunnett’s multiple comparison test. For time-lapse imaging of microglia process rearrangement, a Mann–Whitney test is used to determine significant differences in fluorescence variation. For NMR metabolomic analysis supervised partial least square (PLS) analysis have been carried out on the auto scaled data, building the model in respect to predictors Y, in order to identify the significant variables for the categorization. For the model validation, a full-cross-validation method has been applied, employing as diagnostic statistics *R*^2^, which is a measure of data fitting, *Q*^2^, which is the measure of predictive relevance of the model and the receiver operator characteristics (ROC) values, which measures the specificity and sensitivity of the model.

In order to identify the most significant variables for the model, the variables important in projection (VIP) indexes have been inspected. Variables with a VIP score greater than 1 are considered important for the projection of the PLS regression model [95]. Statistics was carried out employing MatLab 2023a (the MathWorks, Natick, MA) employing in house written functions.

### Reporting summary

Further information on research design is available in the Nature Research Reporting Summary linked to this article.

### Supplementary information

Supplementary figure legends

FigureS1

FigureS2

FigureS3

FigureS4

FigureS5

Reporting summary

## Data Availability

The data analyzed during this study are included in this published article and the Supplementary Data files. Additional supporting data are available from the corresponding authors upon reasonable request.
