# Supplementary material for: Antibiotics treatment promotes vasculogenesis in the brain of glioma-bearing mice
Source: Cell Death Dis. 2024 Mar 13;15(3):210. doi: 10.1038/s41419-024-06578-w (PMC10937980; doi:10.1038/s41419-024-06578-w)
Supplement: Supplementary file 1 — Supplementary figure legends [file 41419_2024_6578_MOESM1_ESM.docx]

**Fig. S1: Tumor growth and microglia frequency in tumor bearing hemisphere**

**(A)** Study design. **(B)** Tumor size in GM and GM/ABX WT mice, *n* = 6, pooled from two experiments, three animals per group. Data are presented as the mean ± SD *p <0.05, Student’s t-test. Upper pictures: Representative images of brain coronal slices, scale bar = 1 mm. **(C)** Tumor size in GM and GM/ABX Cx3cr1^+/gfp^ mice, n = 5 animals per group. Data are presented as the mean ± SD *p <0.05, Student’s t-test. Upper pictures: Representative images of brain coronal slices, scale bar = 1 mm **(D)** Left panel: Flow cytometry quantification of microglia identified as CD45^+^CD11b^+^TMEM119^+^cells in GM and GM/ABX tumoral hemisphere. n=5 mice per group. Data are presented as the mean ± SD**.**

Right panel: Representative gating strategy used to identify microglia by flow cytometry in brain processed samples. First, debris were gated out in the FSC (forward scatter) versus SSC (side scatter) plot; then, singlets, live and CD45^+^ cells were considered; microglia was defined as CD45^+^ CD11b^+^ TMEM119^+^ and shown as percentage among CD45^+^ cells. Numbers in the plots indicates percentage of gated cells in respect to parent cells.

**Fig. S2. Tumor growth in CT2A bearing mice**

**(A)** Tumor size in GM and GM/ABX mice (CT-2A cell line), n = 5 animals per group, pooled from two experiments. Data are presented as the mean ± SD *p <0.05, Student’s t-test. Upper pictures: Representative images of brain coronal slices, scale bar = 1 mm. **(B)** Representative z-projection confocal images of CD34 (magenta) in GM and GM/ABX tumor core area. Hoechst staining (blu) for nuclei visualization; scale bar: 50 μm. **(C)** Scatter dot plots showing quantification of CD34 signal expressed as the percentual area occupied by fluorescent CD34 staining in GM (n= 14/3 slices/mice) and GM/ABX (n= 17/3 slices/mice) mice versus total field of view (FOV). Data are presented as the mean ± SD * p <0.05, Student’s t-test.

**Fig. S3. Representative gating strategies**

Gating strategies used to identify microglia and infiltrating leukocytes by flow cytometry in brain-processed samples. First, debris were gated out in the FSC (forward scatter) vs SSC (side scatter) plot; then, singlets, live and CD45^+^ cells were considered; microglia was defined as CD45^int^ while infiltrating leukocytes as CD45^high^; finally, CD34^+^ cells were evaluated as percentage. Numbers in the plots indicate the percentage of gated cells in respect to parent cells.

**Fig. S4. Metabolome spectra**

**(A)** ^1^H NMR spectrum of fecal water from a control mouse. **(B)** Superimposed fecal water spectra from a control (black) and ABX treated (red) mice. In the insets are highlighted the spectra of Gly-derivative, choline and SCFA; **(C)** ^1^H NMR spectrum of brain extract from a control mouse.

**Fig. S5. Proliferative effect of glycine on GL261 cells**

**(A)** GL261 proliferation analysis by MTT assay after treatment with Glycine at the indicated concentrations and time points. **(B)** GL261 analysis of BrdU positive cells after Glycine treatment. Data are expressed as the number of BrdU+ cells (gray bars) within the total counted cells (white bars).

**Supplementary Table 1. ^1^H NMR assignment of mice’s fecal water**

| Supplementary Table 1: ^1^H NMR assignment of mice’s fecal waters. | | | | |
| --- | --- | --- | --- | --- |
| **Metabolites** | **^1^H δ (ppm)** | **Moiety** | **Multiplicity** | **Level of Assignment*** |
| 2-Oxoglutarate (2-Oxoglut) | 2.44 | β-CH_2_ | t | 3 |
|  | 3.02 | α-CH_2_ | t |  |
| 2-Oxoisovalerate (2-OxoIV) | 1.13^§^ | CH_3_, CH_3_’ | d | 2 |
| Acetate (Ac) | 1.92^§^ | CH_3_ | s | 2 |
| Alanine (Ala) | 1.48^§^ | β-CH_3_ | d | 2 |
|  | 3.77 | α-CH | q |  |
| Bile salts 1 (BS1) | 0.68^§^ | CH_3_ | bs | 3 |
| Bile salts 2 (BS2) | 0.70^§^ | CH_3_ | bs | 3 |
| Bile salts 3 (BS3) | 0.72^§^ | CH_3_ | bs | 3 |
| Butyrate (But) | 0.88 | γ-CH_3_ | t |  |
|  | 1.56^§^ | β-CH_2_ | m | 1 |
|  | 2.16 | α-CH_2_ | t |  |
| Caproate (Cap) | 0.86^§^ | ε-CH_3_ | t | 2 |
|  | 1.28 | γ-CH, δ-CH_2_' | m |  |
|  | 1.54 | β-CH_2_ | m |  |
|  | 2.16 | α-CH_2_ | t |  |
| Choline (Chn) | 3.20^§^ | CH_3_, CH_3_’, CH_3_’’ | bs | 3 |
| Ethanol (EtOH) | 1.18^§^ | CH_3_ | t | 2 |
|  | 3.65 | CH_2_ | q |  |
| Formate (For) | 8.46^§^ | CH | s | 2 |
| Fumarate (Fum) | 6,52^§^ | CH=CH | s | 2 |
| Glutamate (Glu) | 2.04 | β-CH | m |  |
|  | 2.11 | β-CH’ | m |  |
|  | 2.35^§^ | γ-CH_2_ | pt | 2 |
|  |  |  |  |  |
| Glycine-derivative (Gly-derivative) | 3.60^§^ | CH_2_ | s | 3 |
| Hypoxanthine (Hyp) | 8.19 | CH | s |  |
|  | 8.21^§^ | CH | s | 2 |
| Isoleucine (Ile) | 0.95 | CH_3_ | t |  |
|  | 1.01^§^ | CH_3_’ | d | 2 |
|  | 1.26 | γ-CH | m |  |
|  | 1.48 | γ-CH’ | m |  |
|  | 1.96 | β-CH | m |  |
| Lactate (Lac) | 1.33 | CH_3_ | d |  |
|  | 4.11^§^ | CH | q | 2 |
| Leucine (Leu) | 0.97^§^ | CH_3_, CH_3_’ | pt | 2 |
|  | 1.70 | β-CH_2_, γ-CH | m |  |
| Lysine (Lys) | 1.46 | γ-CH_2_ | m |  |
|  | 1.70^§^ | δ-CH_2_ | m | 3 |
|  | 1.90 | β-CH_2_ | m |  |
|  | 3.02 | ε-CH_2_ | pt |  |
|  | 3.75 | α-CH | m |  |
| Methanol (MeOH) | 3.36^§^ | CH_3_ | s | 2 |
| Nicotinate (NA) | 7.52 | CH | t |  |
|  | 8.25 | CH | d |  |
|  | 8.61 | CH | d |  |
|  | 8.94^§^ | CH | bs | 2 |
| Phenylalanine (Phe) | 7.33-7.43^§^ | 1-5 CH | m | 3 |
| Propionate (Prop) | 1.05 | β-CH_3_ | t |  |
|  | 2.17^§^ | α-CH_2_ | q | 1 |
| Succinate (Succ) | 2.41^§^ | α-CH_2_, β-CH_2_ | s | 3 |
| Trymethylamine (TMA) | 2.91^§^ | CH_3_, CH_3_’, CH_3_’’ | s | 3 |
| Tyrosine (Tyr) | 6.90 | 2-CH, 6-CH | d |  |
|  | 7.20^§^ | 3-CH, 5-CH | d | 3 |
| Uracil (U) | 5.80 | CH | d | 2 |
|  | 7.55^§^ | CH | d |  |
| Valine (Val) | 0.99^§^ | CH_3_ | d | 2 |
|  | 1.04 | CH_3_’ | d |  |
|  | 2.26 | β-CH | m |  |
|  | 3.60 | α-CH | d |  |
| α-Glucose (α-Glc) | 3.52 |  | m |  |
|  | 3.66 |  | m |  |
|  | 5,24^§^ | 1-CH | d | 2 |
| β-Arabinose (β-Ara) | 3.53 |  |  |  |
|  | 3.65 |  |  |  |
|  | 3.92 |  |  |  |
|  | 4.53^§^ | 1-CH | d | 3 |
| β-Galactose (β-Gal) | 3.50 |  |  |  |
|  | 3.65 |  |  |  |
|  | 3.95 |  |  |  |
|  | 4.59^§^ | 1-CH | d | 3 |
| β-Xylose (β-Xyl) | 3.24 |  |  |  |
|  | 3.44 |  |  |  |
|  | 3.62 |  |  |  |
|  | 4.57^§^ | 1-CH | d | 3 |
|  |  |  |  |  |
| Keynotes: |  |  |  |  |
| •       Integrated moiety ^§^ |  |  |  |  |
| s: singlet; d: doublet; pd: pseudo-doublet; t: triplet; bs: broad singlet, m: multiplet; dd: doublet of doublets; pt: pseudo-triplet; q: quartet. | | | | |
| * Level 1: identified metabolites; level 2: putatively annotated compounds; level 3: putatively characterized compound classes; level 4: unknown compounds. The level of assignment has been reported according to Salek M. R. et al. doi: 10.1186/2047-217X-2-13 | | | | |

**Supplementary Table 2. ^1^H NMR assignment of mice’s brain extracts**

| Supplementary Table 2: ^1^H NMR assignment of brain exctracts. | | | | |
| --- | --- | --- | --- | --- |
| **Metabolites** | **^1^H δ (ppm)** | **Moiety** | **Multiplicity** | **Level of Assignment*** |
| 3-Hydroxyisobutyrate (3-HIB) | 1,07^§^ | CH_3_ | d | 3 |
|  | 2,52 |  |  |  |
|  | 3,55 |  |  |  |
|  | 3,72 |  |  |  |
| Acetate (Ac) | 1,92^§^ | CH_3_ | s | 2 |
| Adenosine (Ado) | 8,18 | CH | s |  |
|  | 8,34^§^ | CH | s | 3 |
| Alanine (Ala) | 1.48^§^ | β-CH_3_ | d | 2 |
|  | 3.77 | α-CH | q |  |
| Allantoin (All) | 5.38 |  | s |  |
| Aspartate (Asp) | 2.65 | β-CH | dd |  |
|  | 2.80^§^ | β-CH' | dd | 2 |
|  | 3.98 | α-CH | dd |  |
| Adenosine phosphate (AXP) | 8.27 | CH | s |  |
|  | 8.61^§^ | CH | s | 3 |
| Choline (Chn) | 3.20^§^ | CH_3_, CH_3_’, CH_3_’’ | s | 3 |
| Creatine (Crt) | 3.04 | CH_3_ | s |  |
|  | 3.93^§^ | CH_3_ | s |  |
| Cytidine (Cyd) | 6.12 | CH | d |  |
|  | 7.84^§^ | CH | d | 3 |
| Formate (Form) | 8.46^§^ | CH | s | 3 |
| Fumarate (Fum) | 6.53^§^ | CH=CH | s | 3 |
| γ-aminobutyrate (GABA) | 1.90^§^ | β-CH_2_ | m | 2 |
|  | 2.30 | α-CH_2_ | t |  |
|  | 3.02 | γ-CH_2_ | t |  |
| Glutamine (Gln) | 2.13 | β-CH_2_ | m |  |
|  | 2.46^§^ | γ-CH_2_ | m | 2 |
|  | 3.78 | α-CH | m |  |
| Glutamate (Glu) | 2.04 | β-CH | m |  |
|  | 2.11 | β-CH’ | m |  |
|  | 2.35^§^ | γ-CH_2_ | pt | 2 |
|  | 3.75 | α-CH | m |  |
| Glycine (Gly) | 3.56^§^ | CH_2_ | s | 2 |
| Glycerophosphocholine (GPChn) | 3,24^§^ | CH_3_, CH_3_’, CH_3_’’ | s | 3 |
| Guanosine (Guo) | 8.00^§^ | CH | s | 3 |
| Histidine (His) | 7.05 | CH | s |  |
|  | 7,78^§^ | CH | s | 3 |
| Isoleucine (Ile) | 0.95 | CH_3_ | t |  |
|  | 1.01^§^ | CH_3_’ | d | 2 |
|  | 1.26 | γ-CH | m |  |
|  | 1.48 | γ-CH’ | m |  |
|  | 1.96 | β-CH | m |  |
| Lactate (Lac) | 1.33 | CH_3_ | d |  |
|  | 4.11^§^ | CH | q | 2 |
| Leucine (Leu) | 0.97^§^ | CH_3_, CH_3_’ | pt | 2 |
|  | 1.70 | β-CH_2_, γ-CH | m |  |
| Myo-Inositol (Myo-Ino) | 3.27 | 4-CH | t |  |
|  | 3.52^§^ | 2-CH, 6-CH | dd | 2 |
|  | 3.60 | 3-CH,5-CH' | t |  |
|  | 4.05 | 1-CH | pt |  |
| N-Acetyl Aspartate (NAcAsp) | 2.03^§^ | CH_3_ | s | 2 |
|  | 2.50 | α'-CH | dd |  |
|  | 2.70 | α'-CH' | dd |  |
|  | 4.40 | α-CH | dd |  |
| Nicotinamide adenine dinucleotide+ (NAD+) | 8.83 | CH | d |  |
|  | 9.14 | CH | d |  |
|  | 9,33^§^ | CH | bs | 3 |
| Nicotinamide adenine dinucleotide phosphate+ (NADP+) | 8,82 | CH | d |  |
|  | 9,11 | CH | d |  |
|  | 9,3 | CH | bs | 3 |
| Nicotinate (NA) | 7.52 | CH | t |  |
|  | 8.25 | CH | d |  |
|  | 8.61 | CH | d |  |
|  | 8.94^§^ | CH | bs | 2 |
| Phenylalanine (Phe) | 7.33-7.43^§^ | 1-5 CH | m | 3 |
| Phosphocholine (PChn) | 3,22^§^ | CH_3_, CH_3_’, CH_3_’’ | s | 3 |
| Succinate (Succ) | 2.41^§^ | α-CH_2_, β-CH_2_ | s | 3 |
| Taurine (Tau) | 3,26 | CH_3_ | t |  |
|  | 3,42^§^ | CH_3_ | t | 3 |
| Tryptophan (Trp) | 7.19 | 5-CH | t |  |
|  | 7.27 | 6-CH | t |  |
|  | 7.53 | 7-CH | d |  |
|  | 7.73^§^ | 4-CH | d |  |
| Tyrosine (Tyr) | 6.90 | 2-CH, 6-CH | d |  |
|  | 7.20^§^ | 3-CH, 5-CH | d | 3 |
| U01 | 0,75^§^ |  | s | 4 |
| U02 | 3,47^§^ |  | d | 4 |
| U03 | 7.00^§^ |  | bs | 4 |
| Uridine (Urd) | 5.90 | CH | d |  |
|  | 7.88^§^ | CH | d | 3 |
| Uridine phosphate (UXP) | 5.93-5.97 | CH | d |  |
|  | 8.11^§^ | CH | d |  |
| Valine (Val) | 0.99 | CH_3_ | d | 2 |
|  | 1.04^§^ | CH_3_’ | d |  |
|  | 2.26 | β-CH | m |  |
|  | 3.60 | α-CH | d |  |
|  |  |  |  |  |
|  |  |  |  |  |
| Keynotes: |  |  |  |  |
| •       Integrated moiety ^§^ |  |  |  |  |
| s: singlet; d: doublet; pd: pseudo-doublet; t: triplet; bs: broad singlet, m: multiplet; dd: doublet of doublets; pt: pseudo-triplet; q: quartet. | | | | |
| * Level 1: identified metabolites; level 2: putatively annotated compounds; level 3: putatively characterized compound classes; level 4: unknown compounds. The level of assignment has been reported according to Salek M. R. et al. doi: 10.1186/2047-217X-2-13 | | | | |

**Supplementary Table 3. List of primers**

| Gene name | Primer Sequence 5’- 3’ |
| --- | --- |
| GAPDH_forward | TCGTCCCGTAGACAAAATGG |
| GAPDH_reverse | TTGAGGTCAATGAAGGGGTC |
| Chil3_forward | CAGGTCTGGCAATTCTTCTGAA |
| Chil3_reverse | GTCTTGCTCATGTGTGTAAGTGA |
| Tnf𝛂_forward | GTGGAACTGGCAGAAGAG |
| Tnf𝛂_reverse | CCATAGAACTGATGAGAGG |
| il1𝛽_forward | GCAACTGTTCCTGAACTCAACT |
| il1𝛽_reverse | ATCTTTTGGGGTCCGTCAACT |
| nos2_forward | ACATCGACCCGTCCACAGTAT |
| nos2_reverse | CAGAGGGGTAGGCTTGTCTC |
| Mrc1_forward | CAAGGAAGGTTGGCATTTGT |
| Mrc1_reverse | CCTTTCAGTCCTTTGCAAGT |
| P2ry12_forward | CCTGTCGTCAGAGACTACAAG |
| P2ry12_reverse | GGATTTACTGCGGATCTGAAAG |
| P2ry6_forward | GTGAGGATTTCAAGCGACTGC |
| P2ry6_reverse | TCCCCTCTGGCGTAGTTATAGA |
| Arg1_forward | CTCCAAGCCAAAGTCCTTAGAG |
| Arg1_reverse | AGGAGCTGTCATTAGGGACATC |
| cd86_forward | AGAACTTACGGAAGCACCCA |
| cd86_reverse | GGCAGATATGCAGTCCCATT |
| Vegfa_forward | GATCATGCGGATCAAACCTC |
| Vegfa_reverse | AATGCTTTCTCCGCTCTGAA |
| Mmp9_forward | TAGCTACCTCGAGGGCTTCC |
| Mmp9_reverse | GTGGGACACATAGTGGGAGG |
| cd68_forward | GGGCCATGTTTCTCTTGCAA |
| cd68_reverse | AGTCAGTGGCATGGTGAAGA |
| cd133_forward | TGCCACCCCAACTAGAAGAG |
| cd133_reverse | TCGCAGAGCAACTTTTCCAC |
| Nestin_forward | AGGTGTCAAGGTCCAGGATG |
| Nestin_reverse | AAGGAAGCAGACTCAGACCC |
| cd34_forward | CAGGAGAAAGGCTGGGTGAA |
| cd34_reverse | GTTGTCTTGCTGAATGGCCG |
| Olig2_forward | CCCCAGAACCCGATGATCTT |
| Olig2_reverse | GGTGCTGGAGGAAGATGACT |
| cd44_forward | ACCTTGGCCACCACTCCTAA |
| cd44_reverse | GCAGTAGGCTGAAGGGTTGT |
| Sox2_forward | AGGAGAGAAGTTTGGAGCCC |
| Sox2_reverse | TCTGGCGGAGAATAGTTGGG |
