# Supplementary figures and images for: Antibiotics treatment promotes vasculogenesis in the brain of glioma-bearing mice
Source: Cell Death Dis. 2024 Mar 13;15(3):210. doi: 10.1038/s41419-024-06578-w (PMC10937980; doi:10.1038/s41419-024-06578-w)

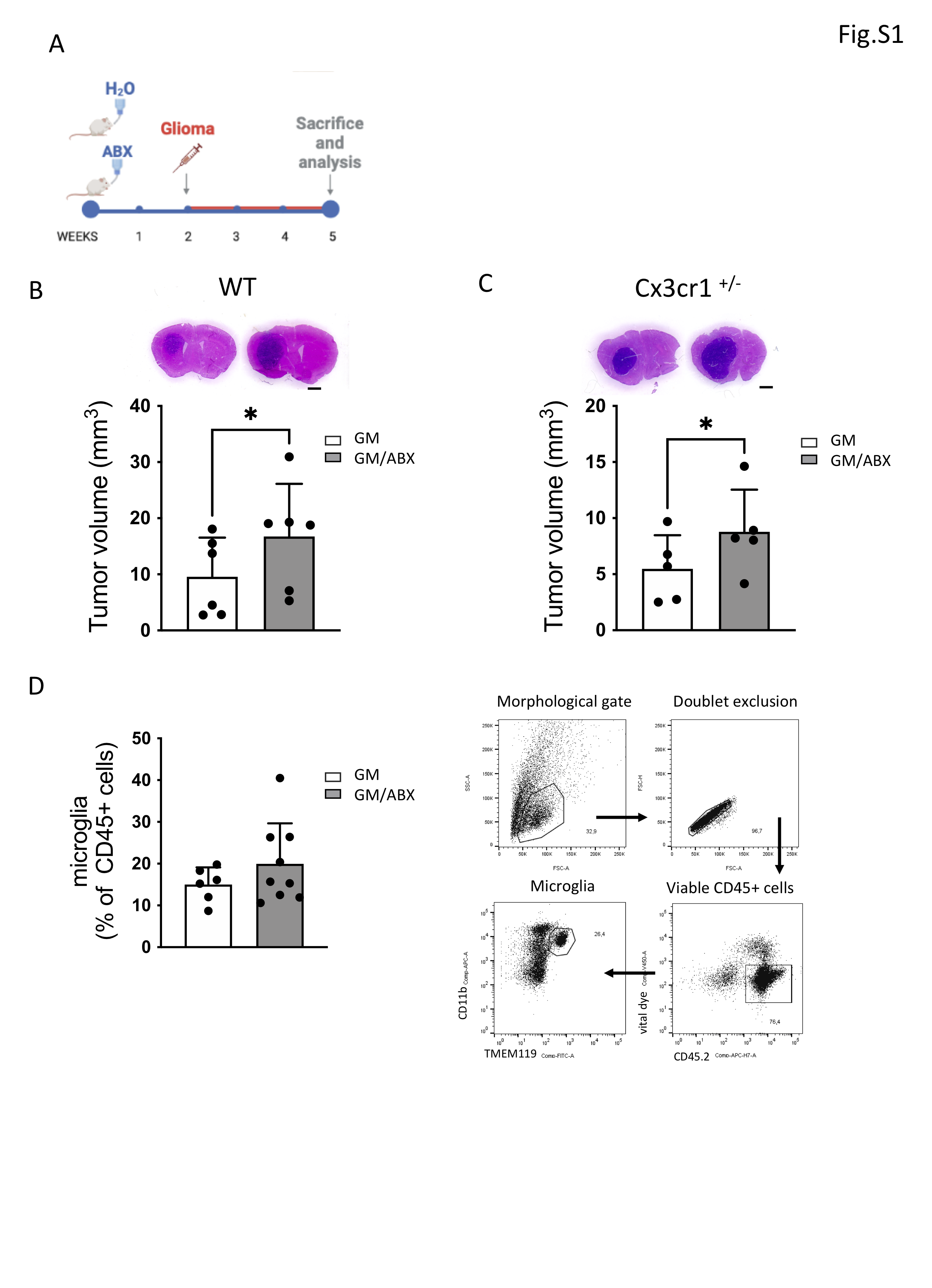

Supplement: Supplementary file 2 — FigureS1 [file 41419_2024_6578_MOESM2_ESM.tif]

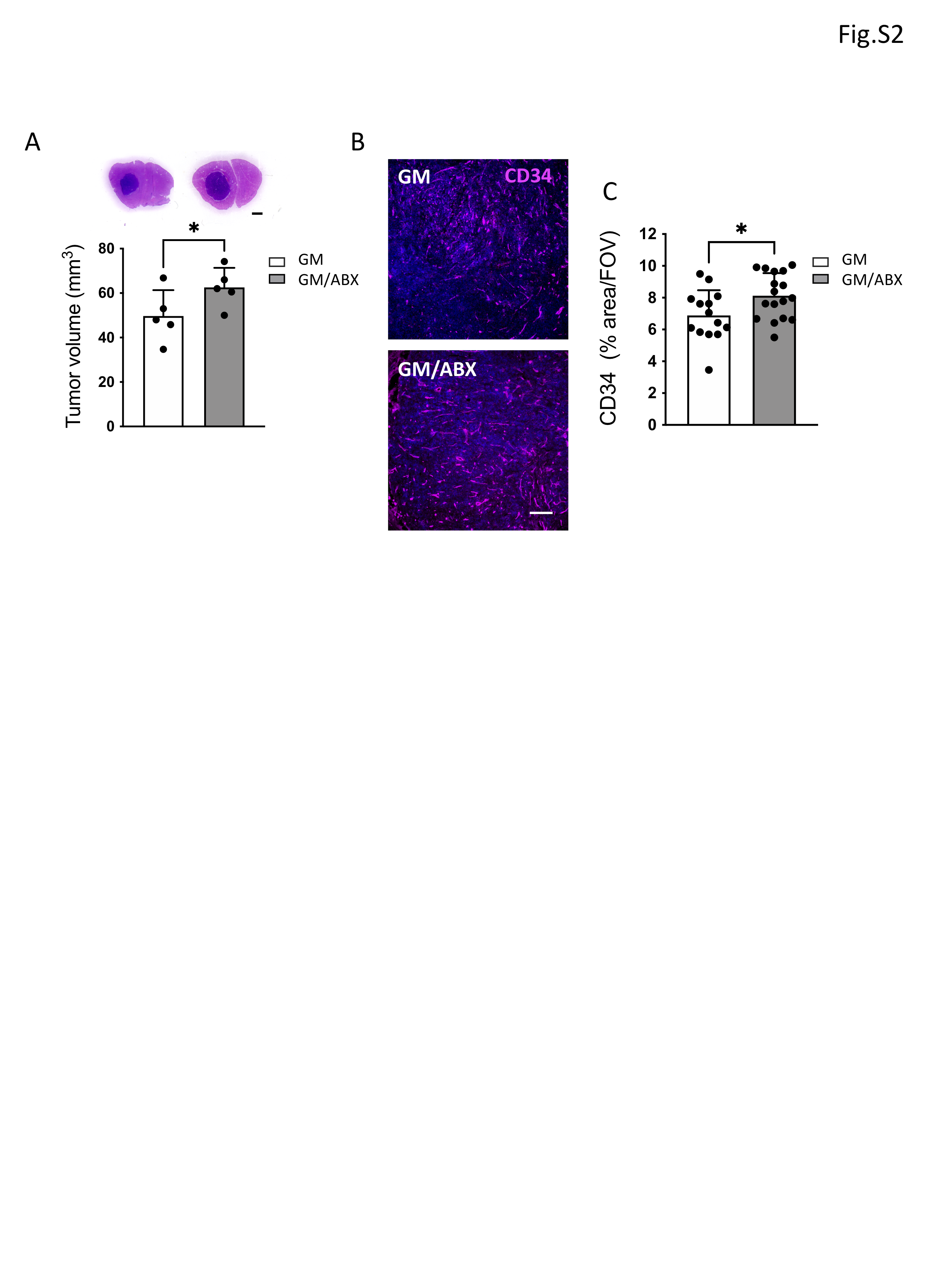

Supplement: Supplementary file 3 — FigureS2 [file 41419_2024_6578_MOESM3_ESM.tif]

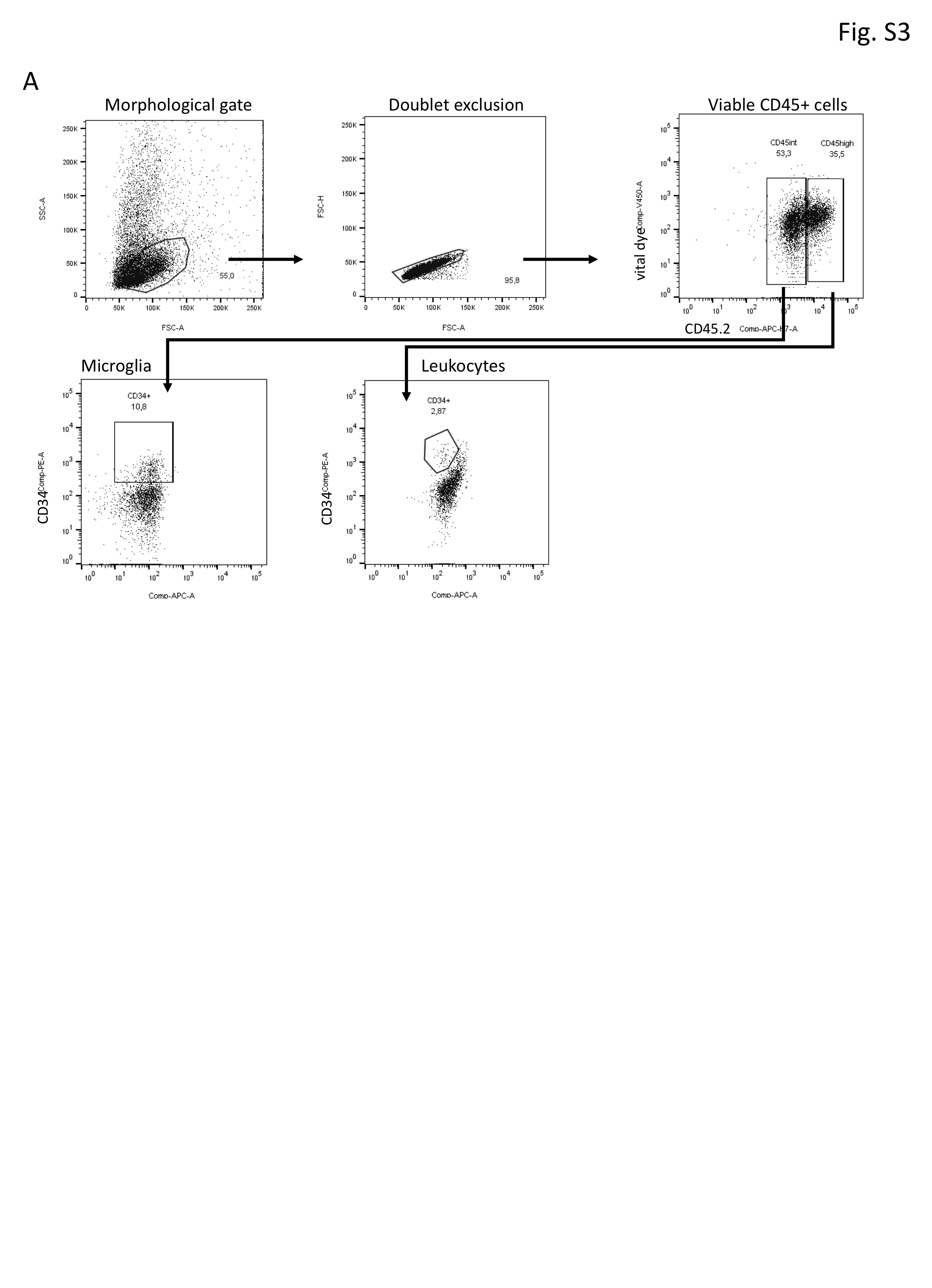

Supplement: Supplementary file 4 — FigureS3 [file 41419_2024_6578_MOESM4_ESM.tif]

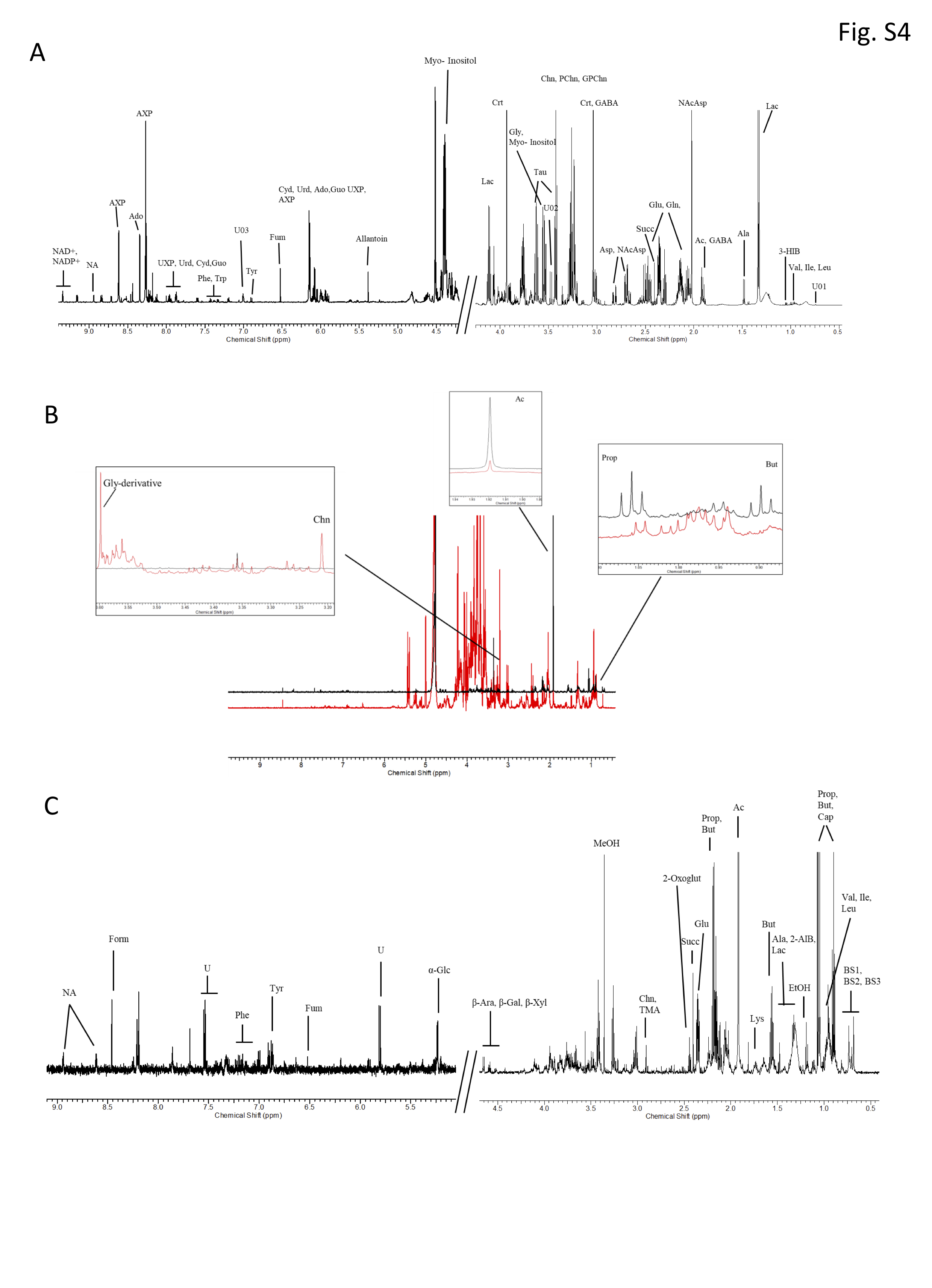

Supplement: Supplementary file 5 — FigureS4 [file 41419_2024_6578_MOESM5_ESM.tif]

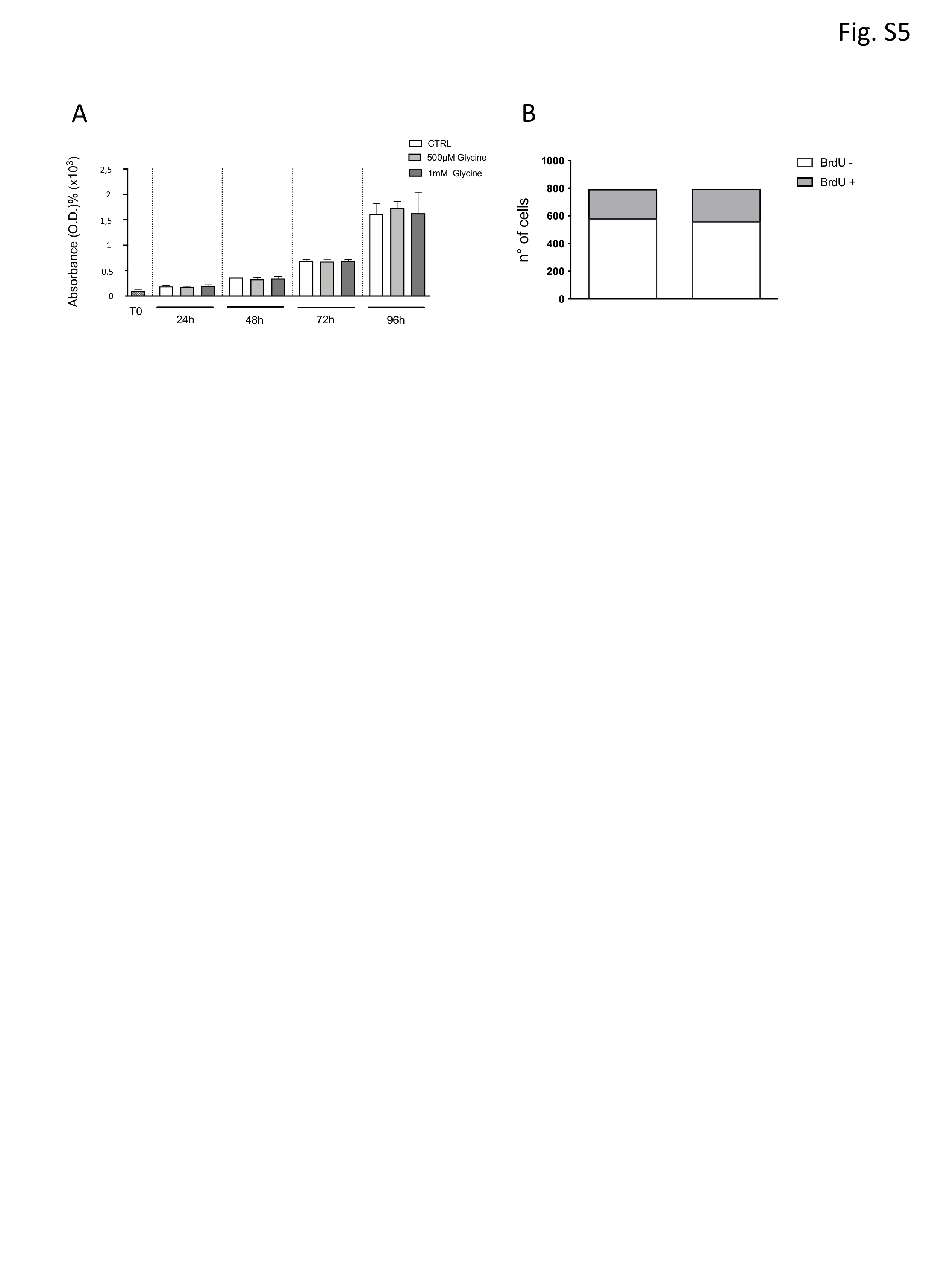

Supplement: Supplementary file 6 — FigureS5 [file 41419_2024_6578_MOESM6_ESM.tif]
